# Don’t blame psychosis, blame the lack of services: a message for early intervention from the Greek standard care model

**DOI:** 10.1186/s12888-022-04212-7

**Published:** 2022-08-22

**Authors:** Stefanos Dimitrakopoulos, Pentagiotissa Stefanatou, Ilias Vlachos, Mirjana Selakovic, Lida-Alkisti Xenaki, Irene Ralli, Rigas-Filippos Soldatos, Nikolaos Nianiakas, Ioannis Kosteletos, Stefania Foteli, Leonidas Mantonakis, Costas T. Kollias, Nikos C. Stefanis

**Affiliations:** 1 First Department of Psychiatry, National and Kapodistrian University of Athens Medical School, Eginition Hospital, 72-74 Vasilissis Sofias Avenue, GR-11528 Athens, Greece; 2 414 Military Hospital of Athens, P. Penteli, Greece; 3 Neurobiology Research Institute, Theodor-Theohari Cozzika Foundation, Athens, Greece

**Keywords:** Athens research study, Early intervention services, First episode psychosis, Relapse, Service engagement

## Abstract

**Background:**

Early Intervention Services (EIS) aim to reduce relapse rates and achieve better treatment and functional outcomes for first episode psychosis (FEP) patients. Existing models of services in Greece are still treatment as usual (TAU), however a reform of mental health services is underway and initial steps have been taken to shift standard care towards EIS. The purpose of the study is to address therapeutic gaps by exploring service engagement and relapse rates in the current standard care model for psychosis.

**Methods:**

We examined follow-up and relapse rates one year after initial treatment contact in the first longitudinal FEP study conducted in Greece. 225 patients were enrolled between 2015–2020. Sociodemographic, clinical and functional characteristics were assessed in association with follow-up and relapse rates.

**Results:**

Within a TAU follow-up setting, one year attrition rates were high. Only 87 patients (38,7%) retained contact with services after one year and within this time frame, 19 of them (21,8%) experienced a severe relapse requiring rehospitalization. Demographic, clinical and functional contributors failed to predict service engagement and relapse rates, with the exception of treatment adherence.

**Conclusion:**

Both follow-up and one-year rehospitalization rates in our FEP sample, highlight the need for the implementation of early intervention services, that will aim at engagement maximization and relapse prevention. These indexes also provide a benchmark against which future early intervention services for psychosis in Greece will have to demonstrate superior efficacy.

## Background

The importance of early intervention in psychosis has been well established [1, 2] and for this purpose Early Intervention Services (EIS) for psychosis have been developed throughout the world [3] aiming at achieving better outcomes and changing the course of the illness [4]. Current literature indicates that favorable long-term outcomes are predicted by achieving symptom remission and functional recovery at the first critical period of psychosis [5]. Researchers argue that relapses over time may not be due to psychosis per se but reflect poor access to mental health services or poor treatment adherence [6]. Principles of reducing duration of untreated psychosis and focusing on family education, supported employment and personalized medication management, are considered as highly important [4, 7]. EIS that are based on the above, in terms of both clinical and functional outcomes, have showed superiority comparing to usual treatment as usual (TAU) Community Care [8].

In recent years, there has been a rising interest in developing such services in Greece [9, 10], based on the international principles of early intervention. For now, existing First-Episode Psychosis (FEP) services in Greece are TAU, mostly hospital-based outpatient services and not meeting EIS standards and for this reason a national strategic approach has been proposed in order to reform our country’s Mental Health System and achieve better long-term benefits for the patients and their families [11]. Under the framework of implementing and organizing EIS, the Athens FEP Research study has been conducted to address treatment outcomes in FEP patients [12]. Conducting FEP studies in our country might elucidate current national mental health needs, providing a benchmark for future reform changes. The purpose of this study is to identify existing therapeutic gaps and needs, by exploring service engagement and relapse rates in a FEP patient cohort, one year after first treatment contact. Moreover, associations of service engagement and relapse rates with possible clinical, sociodemographic and functional contributors are further explored.

## Methods

### Participants

The organizational framework of Athens FEP study has been extensively described elsewhere [12]. Between 2015–2020, 225 patients, from 5 different psychiatric hospitals across Athens, aged 16–45, have been enrolled and have provided inform consent. The inclusion criteria concern ICD-10 diagnosis of drug-induced psychosis (F1x.5), non-affective psychosis (F20-F29), affective psychosis (F30-F33) (WHO, 1992) in patients with first manifestation of psychosis, minimally exposed to antipsychotic medication (less than 2 weeks). Individuals with psychotic symptomatology due to organic causes or acute intoxication, IQ < 65, developmental disorders were excluded. Follow-up was routinely offered in an outpatient basis and information was gathered at the one-year timepoint with a more comprehensive reassessment (by telephone contact or live attendance).

### Psychometric measures and definitions

A number of demographic, clinical and functional variables were collected for analysis as potential predictors of service engagement (vs no service engagement) and relapse (vs no relapse). These included age (age of psychosis onset), gender, education (completed years), diagnosis, social and academic premorbid adjustment, DUP (Duration of Untreated Psychosis), lifetime cannabis use, cognition, presenting symptomatology and functionality both at baseline and one month later, type of hospitalization and length of admission and finally treatment adherence. These specific factors were chosen based both on previously identified predictors of service engagement and relapse [13, 14] as well as their availability in our medical records.

Service disengagement was defined as non-follow-up after multiple contact efforts within a time framework of three months, which is consistent with one of many definitions of service disengagement [13]. Hospitalization is a frequently used proxy for relapse when reporting in a naturalistic setting [15] and in our study relapse was defined by hospitalization due to worsening of psychotic symptomatology during the first year. All subjects were screened using the diagnostic interview for psychosis [DIP] [16] and moreover a consensus diagnosis of two senior psychiatrists was implemented and diagnostic categories were determined [non-affective psychosis (F20-29), affective psychosis (F30-39) and drug-induced psychosis (F10-19)]. The Positive and Negative Symptom Scale [PANSS] [17] was used for the quantitative assessment of symptomatology and dimensions of total, positive, negative and general symptoms scores were assessed both at baseline and one month later. Global Assessment of Functioning [GAF] [18] scale was implemented to evaluate functionality at baseline and one month later. DUP was measured by NOS-DUP scale [19]. Premorbid Adjustment Scale [PAS] [20] was used to define premorbid social and academic adjustment till the age of 15 in order to ensure avoidance of prodromal symptomatology. Cannabis exposure was assessed with Cannabis Experience Questionnaire [CEQ] [21] and a binary variable was constructed by using the cut-off value of once or more per week during the lifetime period of most frequent use [22]. General intellectual capacity was estimated by full scale IQ score using the Greek version of Wechsler Adult Intelligence Scale-fourth edition (WAIS-IV GR) [23, 24]. The type of initial treatment setting was defined as a categorical variable of voluntary hospitalization vs non-voluntary hospitalization vs outpatient visit, while the length of admission was measured as the duration of hospitalization in days. Finally, adherence to medication during the first year was defined as reported by the patients (compliance vs no compliance).

### Statistical analysis

Descriptive statistics are presented for the overall population and stratified by one-year follow-up and moreover, those with 1-year follow up were categorized to those with or without a relapse. All continuous variables are presented with either mean and standard deviation (SD) or median and 1st, 3rd quartiles (Q1, Q3). The association of the one-year follow-up attendance and of relapse at 1st year with demographic, clinical, and functional characteristics was addressed in a univariate level; in case of a categorical variable the Pearson’s chi-squared test was used, while for continuous variables a two-sample t-test or a Mann–Whitney test was applied. As exploratory analyses, the factors or covariates with a *p*-value less than 0.2 in the univariate level, as well as gender and age at onset, were considered for an inclusion in a multiple logistic regression model, from which the results were presented in terms of odds ratios (OR) and 95% confidence intervals (CI). All analyses were performed in the complete case set and were conducted using STATA v.14.2.

## Results

Demographic, clinical and functional characteristics of our sample are presented in Table 1. From the one-year follow-up assessment, only 87 individuals (38,7%) continued to use our FEP services. Univariate analyses did not indicate any association between service engagement at one year follow-up and basic demographic, preclinical, clinical, diagnostic or functional factors (Table 1).

**Table 1:** Overall sample descriptive statistics and one-year service engagement (SE) stratification (1-year SE vs no SE) compared to demographic, clinical and functioning status at baseline and 1-month follow-up, adjusted for age, gender, educational level

Demographics and Diagnosis	With 1-year SE	no SE	*P*	Overall
	***N* = 87**	***N* = 138**		***N* = 225**
**Age of onset according to dup**	***N* = 81**	***N* = 134**		***N* = 215**
Median (Q1—Q3)	23.0 (20.0–31.0)	23.0 (20.0–30.0)	0.844†	23.0 (20.0–31.0)
**Gender, n (%)**	***N* = 87**	***N* = 138**		***N* = 225**
Male	53 (60.9%)	98 (71.0%)	0.117‡	151 (67.1%)
Female	34 (39.1%)	40 (29.0%)		74 (32.9%)
**Education years**	***N* = 85**	***N* = 137**		***N* = 222**
Mean (SD)	13.8 (2.2)	13.6 (2.7)	0.697₸	13.7 (2.5)
**Diagnosis, n (%)**	***N* = 86**	***N* = 138**		***N* = 224**
Non-affective Psychosis	72 (83.7%)	110 (79.7%)	0.151‡	182 (81.3%)
Affective Psychosis	12 (14.0%)	16 (11.6%)		28 (12.5%)
Drug-Induced Psychosis	2 (2.3%)	12 (8.7%)		14 (6.3%)
**Full scale IQ**	***N* = 79**	***N* = 110**		***N* = 189**
Mean (SD)	92.5 (13.0)	92.2 (11.5)	0.579₸	92.3 (12.1)
**Length of admission in days**	***N* = 74**	***N* = 109**		***N* = 183**
Median (Q1—Q3)	33.0 (25.0–42.0)	30.0 (20.0–42.0)	0.147†	31.0 (21.0–42.0)
**Type of hospitalization, n (%)**	***N* = 87**	***N* = 136**		***N* = 223**
Voluntary hospitalization	45 (51.7%)	70 (51.5%)	0.485‡	115 (51.6%)
Unvoluntary hospitalization	32 (36.8%)	43 (31.6%)		75 (33.6%)
Outpatient	10 (11.5%)	23 (16.9%)		33 (14.8%)
**Adherence in medication**	***N* = 61**	***N* = 14**		***N* = 75**
No	13 (21.3%)	1 (7.1%)	0.220‡	14 (18.7%)
Yes	48 (78.7%)	13 (92.9%)		61 (81.3%)
**Lifetime most cannabis use**	***N* = 85**	***N* = 133**		***N* = 218**
Less than one a week	62 (72.9%)	85 (63.9%)	0.165‡	147 (67.4%)
Once and more a week	23 (27.1%)	48 (36.1%)		71 (32.6%)
**PANSS entrance positive symptoms, total scores**	***N* = 87**	***N* = 137**		***N* = 224**
Median (Q1—Q3)	29.0 (24.0–33.0)	27.5 (23.0–32.0)	0.198†	28.0 (23.0–33.0)
**PANSS entrance negative symptoms, total scores**	***N* = 87**	***N* = 137**		***N* = 224**
Median (Q1—Q3)	20.0 (14.0–25.0)	18.0 (13.0–26.0)	0.432†	19.0 (13.5–25.0)
**PANSS entrance general symptoms, total scores**	***N* = 87**	***N* = 137**		***N* = 224**
Median (Q1—Q3)	50.0 (43.0–57.0)	46.0 (37.0–58.0)	0.033†	48.0 (39.0–57.0)
**PANSS entrance total**	***N* = 87**	***N* = 137**		***N* = 224**
Median (Q1—Q3)	99.0 (87.0–114.0)	92.0 (76.0–115.0)	0.055†	94.0 (81.0–114.0)
**PANSS month positive symptoms, total scores**	***N* = 87**	***N* = 136**		***N* = 223**
Median (Q1—Q3)	13.0 (10.0–16.0)	13.0 (11.0–17.5)	0.312†	13.0 (10.0–17.0)
**PANSS month negative symptoms, total scores**	***N* = 87**	***N* = 136**		***N* = 223**
Median (Q1—Q3)	13.0 (9.0–19.0)	12.0 (9.0–18.0)	0.755†	13.0 (9.0–18.0)
**PANSS month general symptoms, total scores**	***N* = 87**	***N* = 136**		***N* = 223**
Median (Q1—Q3)	29.0 (23.0–34.0)	28.0 (24.0–34.5)	0.621†	28.0 (23.0–34.0)
**PANSS month total**	***N* = 87**	***N* = 136**		***N* = 223**
Median (Q1—Q3)	54.0 (45.0–68.0)	56.0 (46.0–67.5)	0.525†	55.0 (45.0–68.0)
**Nottingham DUP (in weeks)**	***N* = 85**	***N* = 138**		***N* = 223**
Median (Q1—Q3)	10.0 (4.0–28.0)	11.0 (5.0–26.0)	0.519†	10.0 (5.0–28.0)
**GAF entrance**	***N* = 82**	***N* = 126**		***N* = 208**
Median (Q1—Q3)	40.0 (30.0–52.0)	35.0 (30.0–50.0)	0.169†	40.0 (30.0–50.0)
**GAF month**	***N* = 80**	***N* = 123**		***N* = 203**
Mean (SD)	60.6 (13.9)	59.6 (13.7)	0.620₸	60.0 (13.7)
**PAS Academic**	***N* = 80**	***N* = 120**		***N* = 200**
Median (Q1—Q3)	0.2 (0.1–0.4)	0.3 (0.1–0.4)	0.470†	0.2 (0.1–0.4)
**PAS Social**	***N* = 80**	***N* = 110**		***N* = 190**
Median (Q1—Q3)	0.2 (0.1–0.4)	0.2 (0.0–0.3)	0.178†	0.2 (0.0–0.3)

*SE* Service Engagement, *PANSS* The Positive and Negative Symptom Scale, *GAF* The Global Assessment of Functioning scale, *DUP* Duration of Untreated Psychosis, *PAS* Premorbid Adjustment Scale

^†^ Mann- Whitney test

^‡^ Pearson’s chi-square test

^₸^ Two-sample t-test with equal variances

At a second level, potential factors from univariate analyses (i.e., *p* < 0.2) were included in a multiple regression model, as well as gender and age, regardless of univariate analyses results. GAF at entrance and PAS Social were excluded due to collinearity with PANSS score. From the exploratory analyses, no association was found between service engagement at one year follow-up and potential factors (Table 2).

**Table 2:** Multiple regression model of one-year service engagement vs no service engagement and clinical factors from univariate analyses (i.e., *p* < 0.2), adjusted for gender and age

no SE vs. 1 yr SE	**Univariate analyses**		**Multiple logistic (*N* = 170)**	
	**OR (95% CI)**	***P***	**OR (95% CI)**	***P***
**Gender (female vs. male)**	0.64 (0.36, 1.12)	0.118	0.71 (0.34, 1.48)	0.364
**Age on onset (years)**	0.99 (0.96, 1.03)	0.748	1.00 (0.96, 1.05)	0.948
**Diagnosis**				
**2 vs. 1**	0.87 (0.39, 1.95)	0.740	1.03 (0.37, 2.85)	0.953
**3 vs. 1**	3.93 (0.85, 18.1)	0.079	3.81 (0.43, 33.7)	0.230
**cannabis use (≥ 1 wk vs. (< 1 wk))**	1.52 (0.84, 2.76)	0.166	1.21 (0.59, 2.48)	0.605
**Length of admission in days**	0.99 (0.97, 1.01)	0.172	0.99 (0.97, 1.01)	0.465
**PANSS entrance total**	0.99 (0.98, 1.00)	0.150	1.00 (0.98, 1.01)	0.786

Only PANSS entrance total was included in the multivariate analysis, as it was collinear with the PANSS entrance general symptoms. The selection of PANSS total score against general symptoms was made under the assumption that total scores represent a more generic profile of study participants and was found to result to an optimal fit regression model

*CI* Confidence interval

A total 19 of 87 who attended at follow-up (21,8%) had experienced relapse with required hospitalization. Univariate analyses did not indicate any association between relapse rates and factors related to PANSS subscales’ score and functionality (at baseline and one-month), diagnosis, DUP, academic and social premorbid adjustment, full scale IQ, cannabis lifetime use, duration and type of hospitalization, with the exception of treatment adherence (Table 3).

**Table 3:** Associations between relapse at first year (relapse vs no relapse) and demographic, clinical and functioning status at baseline and 1-month follow-up, adjusted for age, gender, educational level

Patients with 1 year service engagement Demographics and Diagnosis	Not-relapsed	Relapsed	*P*
	***N* = 68**	***N* = 19**	
**Age of onset according to dup**	***N* = 62**	***N* = 19**	
Median (Q1—Q3)	23.0 (21.0–31.0)	21.0 (18.0–28.0)	0.276†
**Gender, n (%)**	***N* = 68**	***N* = 19**	
Male	41 (60.3%)	12 (63.2%)	0.821‡
Female	27 (39.7%)	7 (36.8%)	
**Education years**	***N* = 67**	***N* = 18**	
Mean (SD)	13.9 (2.2)	13.1 (2.3)	0.164₸
**Diagnosis, n (%)**	***N* = 67**	***N* = 19**	
Non-Affective Psychosis	56 (83.6%)	16 (84.2%)	0.731‡
Affective Psychosis	9 (13.4%)	3 (15.8%)	
Drug-Induced Psychosis	2 (3.0%)	0 (0.0%)	
**Full scale IQ**	***N* = 62**	***N* = 17**	
Mean (SD)	92.2 (13.5)	93.7 (11.2)	0.679₸
**Length of admission in days**	***N* = 57**	***N* = 17**	
Median (Q1—Q3)	34.0 (26.0–41.0)	29.0 (20.0–45.0)	0.782†
**Type of hospitalization, n (%)**	***N* = 68**	***N* = 19**	
Voluntary hospitalization	33 (48.5%)	12 (63.2%)	0.507‡
Unvoluntary hospitalization	27 (39.7%)	5 (26.3%)	
Outpatient	8 (11.8%)	2 (10.5%)	
**Adherrence in medication**	***N* = 47**	***N* = 14**	
No	4 (8.5%)	9 (64.3%)	** < 0.001**‡
Yes	43 (91.5%)	5 (35.7%)	
**Lifetime most cannabis use**	***N* = 66**	***N* = 19**	
Less than one a week	50 (75.8%)	12 (63.2%)	0.276‡
Once and more a week	16 (24.2%)	7 (36.8%)	
**PANSS entrance positive symptoms, total scores**	***N* = 68**	***N* = 19**	
Mean (SD)	29.1 (6.5)	29.6 (7.3)	0.749₸
**PANSS entrance negative symptoms, total scores**	***N* = 68**	***N* = 19**	
Mean (SD)	20.9 (8.0)	19.6 (6.9)	0.524₸
**PANSS entrance general symptoms, total scores**	***N* = 68**	***N* = 19**	
Median (Q1—Q3)	50.0 (42.5–57.0)	50.0 (43.0–57.0)	0.727†
**PANSS entrance total**	***N* = 68**	***N* = 19**	
Median (Q1—Q3)	99.5 (88.5–114.5)	98.0 (86.0–110.0)	0.615†
**PANSS month positive symptoms, total scores**	***N* = 68**	***N* = 19**	
Median (Q1—Q3)	13.0 (10.0–15.5)	12.0 (10.0–18.0)	0.516†
**PANSS month negative symptoms, total scores**	***N* = 68**	***N* = 19**	
Median (Q1—Q3)	13.0 (8.5–19.0)	13.0 (9.0–18.0)	0.984†
**PANSS month general symptoms, total scores**	***N* = 68**	***N* = 19**	
Median (Q1—Q3)	28.5 (23.0–34.0)	30.0 (24.0–35.0)	0.419†
**PANSS month total**	***N* = 68**	***N* = 19**	
Median (Q1—Q3)	52.5 (45.0–67.5)	56.0 (45.0–71.0)	0.565†
**Nottingham DUP (in weeks)**	***N* = 66**	***N* = 19**	
Median (Q1—Q3)	9.5 (4.0–28.0)	10.0 (4.0–29.0)	0.958†
**GAF entrance**	***N* = 63**	***N* = 19**	
Median (Q1—Q3)	40.0 (30.0–55.0)	45.0 (35.0–51.0)	0.223†
**GAF month**	***N* = 61**	***N* = 19**	
Mean (SD)	60.6 (14.2)	60.6 (13.2)	0.984₸
**PAS Academic**	***N* = 62**	***N* = 18**	
Median (Q1—Q3)	0.2 (0.1–0.3)	0.3 (0.1–0.5)	0.462†
**PAS Social**	***N* = 63**	***N* = 17**	
Median (Q1—Q3)	0.2 (0.1–0.4)	0.2 (0.1–0.4)	0.548†

*CI* Confidence interval, *PANSS* The Positive and Negative Symptom Scale, *GAF* The Global Assessment of Functioning scale, *DUP* Duration of Untreated Psychosis, *PAS* Premorbid Adjustment Scale

^†^ Mann- Whitney test

^‡^ Pearson’s chi-square test

^₸^ Two-sample t-test with equal variances

At a second level, potential factors from univariate analyses (i.e., *p* < 0.2) were included in a multiple regression model, as well as gender and age, regardless of univariate analyses results. From the exploratory analyses, only medication adherence was significantly associated with relapse (Table 4).

**Table 4:** Multiple regression model of relapse vs no relapse and clinical, demographic factors from univariate analyses (i.e., *p* < 0.2), adjusted for gender and age

Relapse vs. no relapse	**Univariate analyses**		**Multiple logistic (*N* = 55)**	
	**OR (95% CI)**	***P***	**OR (95% CI)**	***P***
**Gender (female vs. male)**	0.89 (0.31, 2.53)	0.821	1.70 (0.31, 9.21)	0.539
**Age on onset (years)**	1.00 (0.94, 1.07)	0.998	0.94 (0.84, 1.05)	0.266
**Education (years)**	0.84 (0.65, 1.08)	0.166	1.05 (0.71, 1.55)	0.814
**Adherence (yes vs. no)**	0.05 (0.01, 0.23)	** < 0.001**	0.03 (0.004, 0.26)	**0.001**

*CI* Confidence interval

## Discussion

FEP treatment programs are far from implemented and established in our country. In this longitudinal study of FEP patients, the first conducted in Greece, we report rates of relapse within a year from first psychosis manifestation. Moreover, low follow-up rates raise concerns for the efficacy of available FEP Programs and emphasize the need for keeping up with international standards of Service models and the principles of Early Intervention.

In our study, patients who retained contact at one year follow-up time point did not differ in terms of baseline characteristics from FEP patients that refused follow-up, thus they could be considered a representative sample to report relapse rates. Furthermore, this indicates that basic demographic differences or illness-related characteristics are unlikely to explain relapse rates observed within a year of first contact. Relapse within a year was defined stringently by hospital readmission. Relapse rates of 21,8% are consistent with the literature regarding 1^st^ year relapse rates in FEP patients [14]. However, DUP, clinical diagnosis, symptomatology and functionality were not associated with relapse, in contrast with main research findings [4]. The small sample of patients that experienced relapse could be a study limitation and a plausible explanation for the above results. Research evidence suggests that a number of risk factors, predominantly medication non-adherence, substance use disorder and poorer premorbid adjustment have been associated with increased relapse rates [14]. These factors were not found to be related with relapse rates in our analysis with the exception of medication non-adherence as expected [25], however we should consider that we haven’t been able to explore drivers of relapse for individuals who disengaged and this might bias towards FEP patients who followed-up and for whom treatment adherence has a greater impact. Lower relapse rates have been consistently reported as a favourable outcome of EIS vs TAU (19.6% vs 29.1% respectively) [8]. Given that the risk of relapse increases over time [14], we assume that if the follow-up period was extended beyond a year, relapse rates reported in this study may be even higher, similar to comparable TAU studies of longer duration [8]. Furthermore, the high relapse rates reported here cannot be attributable to a broader definition of the construct since relapse was stringently defined as hospitalization rather as a potentially more frequent period of symptom exacerbation. The one-year duration of our follow-up study and the stringent definition of hospitalization as a psychotic symptomatology exacerbation could explain our findings compared to reported rates of 42,4% all-cause hospitalization in TAU services [8].

Low follow-up rates in our study raise skepticism concerning the effectiveness of existing FEP services. While service disengagement is considered a negative prognostic factor [26], the majority of our FEP patients refused continuation of outpatient treatment, as provided by our hospital-based TAU settings. The definition of service disengagement is heterogenous and rates ranging from 6 to 60% [27] and from 12 to 53% [28] are reported. However, recent studies, focusing on EIS, report an outcome of 15,6% on 2 years follow-up average [13] and 32% within the first 12 months after enrollment [29]. In countries where EIS have been established, disengagement rates have declined and the main cause is the multidimensional treatment approach provided to FEP patients [13]. In our study attrition at first year has been 62%, in contrast with reported disengagement rates of studies concerning both EIS and TAU. It is plausible to assume that these high attrition rates could be due to provided treatment, as 4 out of 5 clinical settings across Athens offer TAU as standard follow-up (clinical, hospital-based follow-up without specific psychological or psychosocial interventions). The only FEP Outpatient Service in Athens does not operate within a clear catchment area and it neither provides outreach services to the community nor multidimensional therapeutic approaches based on EIS principles, thus offering services resembling more a TAU setting rather than EIS [9].

Amongst predictors of disengagement, substance use, contact with the criminal justice system, medication non-adherence, lower symptom severity are considered as important [13]. Individuals with low symptom severity are less likely to engage, since they feel they don’t need any treatment, however the clinical profile (as estimated by baseline and first-month PANSS) in our sample was not associated with service engagement. In our analysis, we were not able to explore criminal records or other than cannabis substance use and lack of family support [28] as factors for service disengagement, however, none of the analyzed demographic, clinical- and functionality-related factors were related with service engagement. Given that the greatest challenge for this study is the high drop-out rate, it is hard to determine whether the results represent an accurate description of patients using FEP services in Greece. However, the lack of association with psychosis-related factors explored does not exclude the possibility that other non-specific, non-clinical factors such as the lack of mental health service sectorization, traumatic experiences related to hospitalization or reluctance to be treated with antipsychotic medication might be a reason for not choosing the same clinical setting for follow-up. Engaging a person with the service and building a relationship from which therapy and treatment can be facilitated, is a major contributor for improving outcomes [30]. To address patients’ multidimensional needs, community-based, recovery-oriented, non-stigmatizing assertive programs, such as EIS, might maximize follow-up engagement.

By reporting relapse and high disengagement rates, the Athens FEP study describes the current TAU model of care and underlines the importance for the implementation of EIS in our country providing also a benchmark against which future EIS for psychosis in Greece will have to demonstrate superior efficacy. We propose that a transition from Standard Care to Early Interventions Services would be highly desirable, since they aim at reducing hospital admissions, relapse rates, maximizing follow-up engagement, as in other countries [31]. While we cannot conclusively argue on the specific drivers of relapse rates of patients who disengage, future well designed studies may determine which patients with specific characteristics (clinical, demographic, functional, cognitive) would benefit more from such services. EIS have a clear benefit for patients and their families [8, 32] and moreover there is consistent evidence that the implementation of EIS might be cost-effective for Mental Health Care Systems [33]. While intervention in primary psychosis remains challenging [34, 35], filling the therapeutic gap for FEP patients offering optimized, community-based, multi-disciplinary approaches should be a priority. Quality in Mental Health Services is pivotal for the effectiveness and efficiency of mental healthcare systems [36]. While scarce financial support and lack of government recognition of EIS importance are reported as key barriers for implementation of EIS services [37], a recent government legislation setting the legal framework for the establishment of EIS in Greece (Official Government Gazette of the Hellenic Republic, A 256—23.12.2020) within the National Health System has been an important, but still a first, initial step. Even though there is still much to be done, such advances raise justified optimism for a new era of treating psychosis by implementing fundamental principles of early intervention.

This study has a number of limitations that need to considered. Firstly, the appropriate design that would enable the comparison of outcomes between an EIS vs TAU was not considered, since EIS are substantively unavailable in Greece. The small sample size of patients hospitalized due to a severe relapse could be considered a further study limitation, since the multivariate logistic regression approach is exposed to selection bias. A missing imputation strategy was not followed in the present study, as the reason of missingness was not completely understood and a possible loss of power due to sample size restriction should be considered as a further limitation of our results. Moreover, in our small follow-up sample, unobserved drivers of disengagement and relapse further increase selection bias towards interpreting our findings. Treatment discontinuation could not be distinguished from partial non-adherence, as it is based on patients’ self-report. Likewise, cannabis use variable may also include a reporting bias.

In summary, our FEP study demonstrated that demographic, clinical and functional determinants failed to predict high service engagement and relapse rates during the first year, however we should consider existing methodological limitations and that absence of evidence is unlikely to be evidence of absence. By reporting service engagement and one-year hospitalization rates in our FEP sample, current TAU care model in Greece is described and a baseline is set against which an upcoming health policy concerning future EIS implementation should be judged.

## Data Availability

Data are available from the corresponding author on reasonable request.

## References

[1] McGorry PD, Killackey E, Yung A (2008). Early intervention in psychosis: concepts, evidence and future directions. World Psychiatry.

[2] Malla A, McGorry P (2019). Early intervention in psychosis in young people: a population and public health perspective. Am J Public Health.

[3] Kotlicka-Antczak M, Podgórski M, Oliver D, Maric NP, Valmaggia L, Fusar-Poli P (2020). Worldwide implementation of clinical services for the prevention of psychosis: the IEPA early intervention in mental health survey. Early Interv Psychiatry.

[4] Fusar-Poli P, McGorry PD, Kane JM (2017). Improving outcomes of first-episode psychosis: an overview. World Psychiatry.

[5] Álvarez-Jiménez M, Gleeson JF, Henry LP, Harrigan SM, Harris MG, Killackey E (2012). Road to full recovery: longitudinal relationship between symptomatic remission and psychosocial recovery in first-episode psychosis over 7.5 years. Psychol Med.

[6] Zipursky RB, Reilly TJ, Murray RM (2013). The myth of schizophrenia as a progressive brain disease. Schizophr Bull.

[7] Mueser KT, Meyer-Kalos PS, Glynn SM, Lynde DW, Robinson DG, Gingerich S (2019). Implementation and fidelity assessment of the NAVIGATE treatment program for first episode psychosis in a multi-site study. Schizophr Res.

[8] Correll CU, Galling B, Pawar A, Krivko A, Bonetto C, Ruggeri M (2018). Comparison of early intervention services vs treatment as usual for early-phase psychosis: a systematic review, meta-analysis, and meta-regression. JAMA Psychiat.

[9] Kollias C, Xenaki LA, Dimitrakopoulos S, Kosteletos I, Kontaxakis V, Stefanis N (2018). Early psychosis intervention outpatient service of the 1st Psychiatric University Clinic in Athens: 3 years of experience. Early Interv Psychiatry.

[10] Mantas C, Mavreas V (2012). Establishing and operating an early intervention service for psychosis in a defined catchment area of northwestern Greece within the context of the local mental health network. Early Interv Psychiatry.

[11] Stefanis NC, Mavreas V, Νimatoudis Ι, Gourzis F, Samakouri Μ, Vgontzas A (2018). A proposal for the implementation of Early Intervention in Psychosis (EIP) services in Greece: if not now, when?. Psychiatriki.

[12] Xenaki LA, Kollias CT, Stefanatou P, Ralli I, Soldatos RF, Dimitrakopoulos S (2020). Organization framework and preliminary findings from the Athens First-Episode Psychosis Research Study. Early Interv Psychiatry.

[13] Robson E, Greenwood E. Rates and predictors of disengagement and strength of engagement for people with a first episode of psychosis using early intervention services: a systematic review of predictors and meta-analysis of disengagement rates. Schizophrenia Bulletin Open. 2022;3(1):1-26. 10.1093/schizbullopen/sgac012 PMC11205872 39144778

[14] Alvarez-Jimenez M, Priede A, Hetrick SE, Bendall S, Killackey E, Parker AG (2012). Risk factors for relapse following treatment for first episode psychosis: a systematic review and meta-analysis of longitudinal studies. Schizophr Res.

[15] Olivares JM, Sermon J, Hemels M, Schreiner A (2013). Definitions and drivers of relapse in patients with schizophrenia: a systematic literature review. Ann Gen Psychiatry.

[16] Castle DJ, Jablensky A, McGrath JJ, Carr V, Morgan V, Waterreus A (2006). The diagnostic interview for psychoses (DIP): development, reliability and applications. Psychol Med.

[17] Lykouras E, Botsis A, Oulis P (1994). The Positive and Negative Syndrome Scale (PANSS) [in Greek].

[18] Hall RC (1995). Global assessment of functioning. A modified scale. Psychosomatics.

[19] Singh SP, Cooper JE, Fisher HL, Tarrant CJ, Lloyd T, Banjo J (2005). Determining the chronology and components of psychosis onset: the Nottingham Onset Schedule (NOS). Schizophr Res.

[20] Rabinowitz J, Levine SZ, Brill N, Bromet EJ (2007). The premorbid adjustment scale structured interview (PAS-SI): preliminary findings. Schizophr Res.

[21] Barkus E, Lewis S (2008). Schizotypy and psychosis-like experiences from recreational cannabis in a non-clinical sample. Psychol Med.

[22] Pries LK, Lage-Castellanos A, Delespaul P, Kenis G, Luykx JJ, Lin BD (2019). Estimating exposome score for schizophrenia using predictive modeling approach in two independent samples: the results from the EUGEI Study. Schizophr Bull.

[23] Stogiannidou A (2011). WAIS-IV GR (Wechsler Adult Intelligence Scale).

[24] Wechsler D (2008). Wechsler adult intelligence scale.

[25] Zipursky RB, Menezes NM, Streiner DL (2014). Risk of symptom recurrence with medication discontinuation in first-episode psychosis: a systematic review. Schizophr Res.

[26] Bidargaddi N, Schrader G, Myles H, Schubert KO, van Kasteren Y, Zhang T (2021). Demonstration of automated non-adherence and service disengagement risk monitoring with active follow-up for severe mental illness. Aust N Z J Psychiatry.

[27] Reynolds S, Kim DJ, Brown E, Tindall R, O'Donoghue B (2019). Defining disengagement from mental health services for individuals experiencing first episode psychosis: a systematic review. Soc Psychiatry Psychiatr Epidemiol.

[28] Mascayano F, van der Ven E, Martinez-Ales G, Henao AR, Zambrano J, Jones N (2021). Disengagement from early intervention services for psychosis: a systematic review. Psychiatr Serv.

[29] Mascayano F, van der Ven E, Martinez-Ales G, Basaraba C, Jones N, Lee R (2020). Predictors of early discharge from early intervention services for psychosis in New York State. Psychiatr Serv.

[30] Tindall RM, Allott K, Simmons M, Roberts W, Hamilton BE (2019). The missing voice of engagement: an exploratory study from the perspectives of case-managers at an early intervention service for first-episode psychosis. BMC Psychiatry.

[31] Colizzi M, Lasalvia A, Ruggeri M (2020). Prevention and early intervention in youth mental health: is it time for a multidisciplinary and trans-diagnostic model for care?. Int J Ment Health Syst.

[32] McGorry PD (2015). Early intervention in psychosis: obvious, effective, overdue. J Nerv Ment Dis.

[33] Aceituno D, Vera N, Prina AM, McCrone P (2019). Cost-effectiveness of early intervention in psychosis: systematic review. Br J Psychiatry.

[34] Dimitrakopoulos S, Kollias C, Stefanis NC, Kontaxakis V (2015). Early psychotic experiences: interventions, problems and perspectives. Psychiatriki.

[35] Maj M, van Os J, De Hert M, Gaebel W, Galderisi S, Green MF (2021). The clinical characterization of the patient with primary psychosis aimed at personalization of management. World Psychiatry.

[36] Gaebel W, Kerst A, Janssen B, Becker T, Musalek M, Rössler W (2020). EPA guidance on the quality of mental health services: a systematic meta-review and update of recommendations focusing on care coordination. Eur Psychiatry.

[37] O'Connell N, O'Connor K, McGrath D, Vagge L, Mockler D, Jennings R (2021). Early Intervention in Psychosis services: a systematic review and narrative synthesis of the barriers and facilitators to implementation. Eur Psychiatry.

